# CTELS: A Cell-Free System for the Analysis of Translation Termination Rate

**DOI:** 10.3390/biom10060911

**Published:** 2020-06-16

**Authors:** Kseniya A. Lashkevich, Valeriya I. Shlyk, Artem S. Kushchenko, Vadim N. Gladyshev, Elena Z. Alkalaeva, Sergey E. Dmitriev

**Affiliations:** 1 Belozersky Institute of Physico-Chemical Biology, Lomonosov Moscow State University, 119234 Moscow, Russia; akulichksenia@gmail.com (K.A.L.); ymmo@mail.ru (V.I.S.); artkushchenko@gmail.com (A.S.K.); 2 Department of Molecular Biology, Biological Faculty, Lomonosov Moscow State University, 119991 Moscow, Russia; 3 School of Bioengineering and Bioinformatics, Lomonosov Moscow State University, 119234 Moscow, Russia; 4 Division of Genetics, Department of Medicine, Brigham and Women’s Hospital, Harvard Medical School, Boston, MA 02115, USA; vgladyshev@rics.bwh.harvard.edu; 5 Engelhardt Institute of Molecular Biology, Russian Academy of Sciences, 119991 Moscow, Russia; alkalaeva@eimb.ru

**Keywords:** translation termination, nascent peptide release, firefly luciferase, in vitro translation system, eukaryotic release factors, eRF1, eRF3, eRF1(AGQ) mutant, blasticidin S, 3′ UTR length, stop codon read-through

## Abstract

Translation termination is the final step in protein biosynthesis when the synthesized polypeptide is released from the ribosome. Understanding this complex process is important for treatment of many human disorders caused by nonsense mutations in important genes. Here, we present a new method for the analysis of translation termination rate in cell-free systems, CTELS (for C-terminally extended luciferase-based system). This approach was based on a continuously measured luciferase activity during in vitro translation reaction of two reporter mRNA, one of which encodes a C-terminally extended luciferase. This extension occupies a ribosomal polypeptide tunnel and lets the completely synthesized enzyme be active before translation termination occurs, i.e., when it is still on the ribosome. In contrast, luciferase molecule without the extension emits light only after its release. Comparing the translation dynamics of these two reporters allows visualization of a delay corresponding to the translation termination event. We demonstrated applicability of this approach for investigating the effects of cis- and trans-acting components, including small molecule inhibitors and read-through inducing sequences, on the translation termination rate. With CTELS, we systematically assessed negative effects of decreased 3′ UTR length, specifically on termination. We also showed that blasticidin S implements its inhibitory effect on eukaryotic translation system, mostly by affecting elongation, and that an excess of eRF1 termination factor (both the wild-type and a non-catalytic AGQ mutant) can interfere with elongation. Analysis of read-through mechanics with CTELS revealed a transient stalling event at a “leaky” stop codon context, which likely defines the basis of nonsense suppression.

## 1. Introduction

Translation termination is an important and indispensable step in mRNA translation cycle [1,2]. It is initiated as UAA, UAG, or UGA enters the ribosomal A site, includes stop codon recognition and peptidyl-tRNA hydrolysis. Termination ends up with the nascent peptide release and is followed by ribosome recycling, preparing the ribosome subunits for a new round of translation.

In eukaryotes, termination is governed by two specialized protein factors, eRF1 and eRF3, operating in a complex with GTP [3,4]. eRF1 molecule structurally mimics tRNA. It requires a highly conserved GGQ loop for posing a water molecule to perform peptydyl-tRNA hydrolysis [5,6,7,8]. eRF3 is a translational GTPase, which is thought to deliver eRF1 to the ribosome [9,10]. By interacting with many protein partners (including poly(A)-binding protein (PABP), nonsense-mediated decay (NMD) factors, components of mRNA deadenylation machinery and many others), it is also responsible for coupling termination with many other processes of RNA metabolism, like mRNA quality control, functional cyclization, localization, and decay (for review, see [11,12,13]).

Recent structural studies revealed a peculiar molecular architecture of the eukaryotic pre-termination complex [7,8,14,15,16]. In particular, during stop codon recognition, binding of eRF1 flips nucleotide A1825 of 18S ribosomal RNA so that it stacks on the second and third bases of a stop codon. This results in pulling one downstream nucleotide into the A-site, where it forms a specific U-turn structure with three stop codon bases and contributes to discrimination against sense codons [7,8,14]. The U-turn formation leads to retraction of the mRNA 3′ end to the entry channel, which was detected earlier by toe-printing analyses and ribosome profiling [10,17]. Importantly, binding of the eRF1-eRF3-GTP complex does not cause peptide release immediately, because the catalytic GGQ loop is initially positioned too far from the peptidyl transferase center. Recognition of stop-codon by eRF1 changes conformation of eRF3, triggering GTP hydrolysis. As a result, eRF3 leaves or changes its position, forcing the eRF1 GGQ loop to move toward the peptidyl-tRNA and catalyze its hydrolysis [14,15,18]. Amino acid substitutions in the GGQ loop disables hydrolytic activity of eRF1, e.g., the eRF1(AGQ) mutant is unable to release the protein, while it can still bind to stop codon [5,6]. The Gln side chain in GGQ is methylated, a feature conserved from bacteria to human, facilitating its function [19].

eRF1 and eRF3 activity can also be tuned by many other translation factors and regulators, including proteins bound to mRNA poly(A)-tail. PABP directly interacts with eRF3 and stimulates termination, while PAIP1 and PAIP2 modulate these effects (see [20,21,22,23] and references therein). Many other auxiliary proteins, such as the ABCE1/Rli1p, eIF5A, DDX19/Dbp5p, Gle1, and NMD components, are implicated in peptidyl-tRNA hydrolysis or control translation termination [11,12,13,20,23,24,25,26,27,28,29,30,31]. Cis-acting factors can also affect termination efficiency. Under normal conditions, at the majority of stop codons termination occurs effectively, providing a fidelity of ~99% (see, for example, [32]). However, “leaky” nucleotide contexts of stop codon highly elevate the read-through level (see [33,34,35,36] and references therein). Some viruses actively adopted translational read-through in their normal life [37,38]. For example, Venezuelan equine encephalitis virus (VEEV) evolves a developed secondary structure after stop codon to impair translation termination [34,39].

Investigation of translation termination is important for the development of targeted therapy, as more than 10% of hereditary diseases, as well as cancer cases are caused by premature termination codons (PTC), leading to the synthesis of truncated nonfunctional proteins and NMD-mediated degradation of the mRNA templates [40,41]. Nonsense-suppression therapeutics is aimed to stimulate incorporation of a near-cognate aminoacyl-tRNA to provide synthesis of full-length protein by enhancing the read-through rate on the PTCs [42,43]. The appropriate drug could potentially be used to treat a large number of hereditary diseases at once. However, a few inhibitors are known to interfere specifically with translation termination [44,45,46]. The most promising are probably aminoglycoside antibiotics, the drugs inducing misincorporation of amino acids (see [47,48,49] and references therein). Taken in appropriate concentrations, they can induce read-through at PTCs, although at the same time reduce translation fidelity, which might cause nephrotoxicity and ototoxicity during long-term treatments [49,50,51,52]. Blasticidin S was reported to strongly inhibit peptidyl-tRNA hydrolysis in bacteria by expelling the RF1 GGQ motif from the peptidyl transferase center [53,54]. However, blasticidin S was initially described as peptidyl-transferase inhibitor (for review, see [44]), so its potential as a specific termination inhibitor, especially in eukaryotes, is controversial. A number of less characterized small molecules were also reported to affect this stage [55,56,57,58,59,60,61,62,63], but some of them have been studied in bacterial systems only, some are not yet commercially available, or have an obscure mechanism of action, or already failed in clinical trials [42,64,65,66].

To investigate translation termination and to assess its efficiency, several approaches have been developed. To measure read-through rate, reporter genes with PTC are used. In the most common versions, coding regions of two different luciferase or fluorescent proteins, present on the same transcript, are separated by a stop codon, so the ratio of the two products can be taken to evaluate read-through efficiency [67,68]. Such approaches are very convenient for analyzing the efficiency of read-through events, but tell nothing about the kinetics and molecular mechanics of the process. Ribosome profiling was recently applied to study termination and stop codon suppression [48,69,70,71,72,73] and revealed pervasive ribosome occupancy of mRNA regions downstream of stop codons, likely due to defective or regulated ribosome recycling, reinitiation, mRNA degradation, or other processes [74,75,76,77,78,79,80,81]. However, it is also hardly used for analysis of translation dynamics. A wide arsenal of biochemical approaches has been applied to study translation termination in reconstituted systems, including toe-printing [10,82] and radiolabeled Met-tRNA hydrolysis or peptide release [10]. Although much important information has been obtained with these methods, purified ribosomes and translation factors poorly recapitulate situation in complete system or in living cells. Thus, other approaches are needed to study molecular mechanisms in complete cell lysates or in vivo.

In this work we propose the principally new method for estimating the termination rate in a cell-free system, based on a C-terminally extended luciferase-based system (CTELS). This approach uses a combination of specially designed mRNA constructs. Comparison of their translation kinetics enables detection of translation defects and attributes them specifically to the stage of termination. Using CTELS, we evaluated the effects of small molecule drugs, translation factors, 3′ UTR length and read-through-promoting sequences on the translation termination rate. These findings allow us to propose a stalling-induced model of nonsense suppression.

## 2. Materials and Methods

### 2.1. Plasmid Constructs and In Vitro Transcription

All plasmids were prepared on the basis of the pGL3R-β-glo plasmid [83]. To obtain the CTELS1 construct, a two-step PCR was performed—first, two PCR products were obtained from the pGL3R-β-glo plasmid with primers FlucExt1F and FlucExt1F, FlucExt2F and FlucExt2R (for a complete list of plasmids and primers used, see Supplementary Materials, Tables S1 and S2); then these partially overlapped products were mixed and used as a template for second PCR with primers FlucExt1F and FlucExt2R. The resulting product was digested by DraII and HpaI and inserted into the original pGL3R-β-glo treated by the same enzymes. This duplicated the last 150 nt of the luciferase coding region and also introduced EcoRI site between the repeated parts. The plasmid CTELS2 was obtained from CTELS1 by inserting a fragment of the human *HBB* cDNA (amplified with primers GloExtF and GloExtR) into the CTELS1 backbone, after their treatment with EcoRI and HpaI.

PCR products amplified from the plasmids with T7Glo_F and the corresponding reverse primers (see Table S2) were used as templates for the mRNA synthesis, essentially as described previously [84]. Reverse primers with 50T at the 5′ terminus were taken to obtain polyadenylated transcripts. As the 3′ terminus of luciferase coding region was duplicated in the CTELS1 plasmid, it was impossible to use reverse primers annealing to this region for preparation of transcription templates in this case. In contrast, the 3′ proximal part of the CTELS2 coding region was represented by a unique sequence, this enabled the generation of transcription templates with variable 3′ termini (e.g., to append various 3′ UTR sequences), simply by changing a reverse primer. For transcription, the RiboMAX kit (Promega) was used. The resulting transcripts were precipitated with 2M LiCl, then capped with Vaccinia Capping System (NEB) and precipitated with LiCl, once again. All mRNA transcripts were checked for integrity by denaturing urea polyacrylamide gel electrophoresis.

### 2.2. Recombinant Proteins Expression and Purification

Human release factor eRF1 and its mutant form (eRF1(AGQ), were produced as recombinant proteins in E. coli strain BL21 and subsequently purified via Ni-NTA agarose and ion-exchange chromatography, as described previously [5]. The proteins were stored in frozen aliquots in buffer A100 (20 mM Tris-HCl, pH 7.4, 100 mM KCl, 10% glycerol, 1 mM DTT).

### 2.3. In Vitro Translation in Mammalian Cell-Free Systems

Nuclease-treated rabbit reticulocyte lysate (RRL) was purchased from Promega (#L4960) and used in accordance with the supplier’s recommendation. The total reaction volume was 15 μL and contained 7.5 μL of the lysate, 3 u of RiboLock RNase inhibitor (Thermo Fisher Scientific), 0.5 mM D-luciferin, and 100 ng mRNA. S30 extract of the Krebs-2 mouse ascite cells was prepared, as described previously [83]. Translation reactions in the Krebs-2 S30 system were performed in a total volume of 15 μL, containing 7.5 μL of the lysates, 1× Translation Buffer (20 mM Hepes-KOH pH 7.4, 1 mM DTT, 0.5 mM spermidine-HCl, 1 mM Mg(OAc)_2_, 8 mM creatine phosphate, 1 mM ATP, 0.2 mM GTP, 120 mM KOAc, and 25 μM of each amino acid), 3 u of RiboLock RNase inhibitor, 0.5 mM D-luciferin and 100 ng mRNA. In the case of the hybrid RRL/Krebs-2 system, the reaction was performed in 15 μL, which contained 7.5 μL of RRL, 3 μL of S30, 3 u of RiboLock RNase inhibitor, 0.5 mM D-luciferin, and 100 ng mRNA. In experiments with recombinant proteins, 1 μL of protein solution in buffer A100, or just the buffer, were added, as indicated. In experiments with blasticidin S HCl (Thermo Fisher Scientific), 1 μL of 15× solution in water was added. After mRNA addition, the mixtures were transferred into the pre-heated white FB/NB 384-well plate (Grenier #781904), covered by a PCR plate seal and incubated in the CLARIOstar plate reader (BMG Labtech) at 30 °C, with continuous measurement of the luciferase activity (integration time 1 s), essentially as described [85]. Time intervals between loading the samples into plate wells were measured and taken into account when plotting curves. All experiments were repeated 3–5 times, representative curves are shown.

## 3. Results

### 3.1. Development of a Luciferase-Based Cell-Free System for the Analysis of Rate and Efficiency of Translation Termination

Cell-free translation systems with real-time monitoring of a synthesized product are extensively used for investigating various aspects of protein biosynthesis [86,87,88,89]. Firefly luciferase, a light producing enzyme from *Photinus pyralis*, perfectly fits this approach, due to the absence of post-translational modification and ease of detection. This 62 kDa protein was shown to be completely folded and thus enzymatically active, immediately upon release from the ribosome in both plant and mammalian cell-free systems [86,90]. Moreover, when its C-terminus was extended by at least 26 additional amino acid residues, it acquired the enzymatic activity even before the release, i.e., in the ribosome-bound form [91].

**Figure 1:**
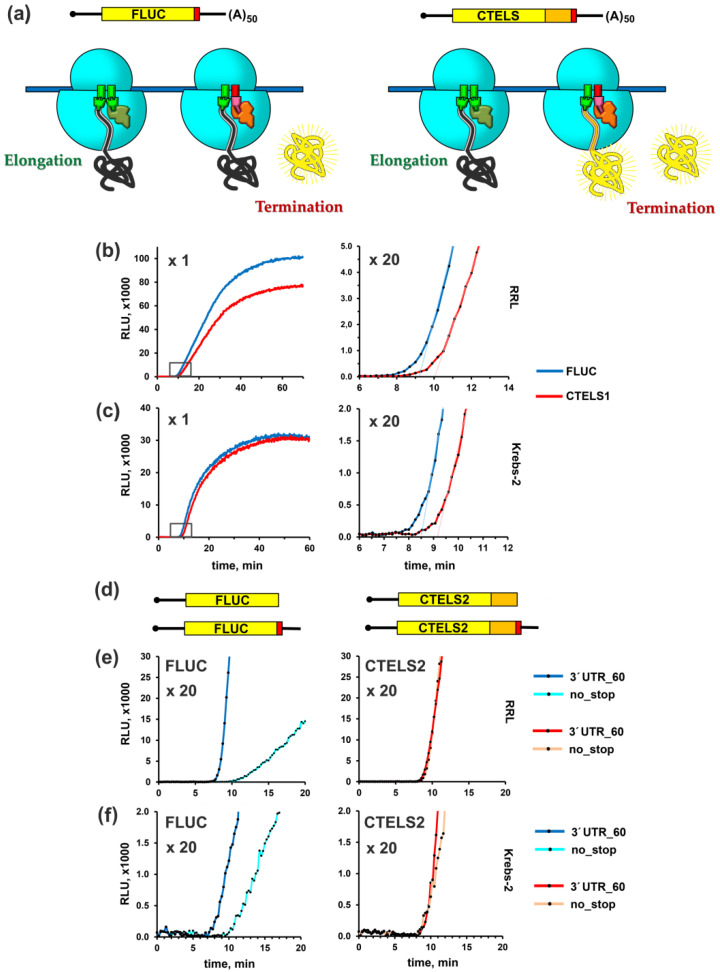
Development of CTELS—a C-terminally extended luciferase-based system—for the analysis of rate and efficiency of translation termination. (**a**) Schematic representation of a pair of FLUC and CTELS reporter mRNAs and their products on the ribosome. Conventional luciferase (FLUC) is inactive until its release from the ribosome, as its C-terminus is held within the ribosome peptide tunnel (on the left, black vs. yellow). Luciferase with a 50 amino acid extension (shown in orange) becomes active while still being attached to the ribosome, as the active luciferase enzyme is co-translationally folded outside, while the tunnel in filled by the extension. Stop codon is shown in red. (**b**,**c**) Time-course of luciferase activity accumulation in a translation reaction programmed by capped and polyadenylated transcripts, encoding firefly luciferase with and without the C-terminal extension (CTELS1, red curve, and FLUC, blue curve, respectively) in two different cell-free systems, nuclease-treated RRL (**b**) and S30 extract of the Krebs-2 mouse ascite cells (**c**). Luminescence was continuously measured with the integration time of 1 s. The right panels represent the boxed areas from the left panels in an increased scale. Dashed lines show the mean slopes of the linear fit of the data collected at 10–15 min. Representative curves obtained in one out of three independent experiments are shown. (**d**) mRNA constructs (FLUC- and CTELS2-based, with and without stop-codon and the 60-nt long (agcatc)_10_ 3′ UTR) used to confirm the ability of CTELS to produce luciferase activity before the product is released from the ribosome. (**e**,**f**) Luciferase activity produced by translation of the FLUC- and CTELS2-based mRNAs in RRL (**e**) and Krebs-2 S30 extract (**f**), shown in an increased scale.

We reasoned that these features of the enzyme could be used for monitoring the physiological polypeptide release from the ribosome during translation termination. To this end, we developed a system of two similar reporter mRNAs, one of which encodes the original luciferase (hereafter denoted as FLUC construct), while another produced a C-terminally extended luciferase. We called it CTELS, for C-terminally extended luciferase-based system (Figure 1a).

In the first variant of CTELS, the extension was made by repeating the last 50 amino acids of luciferase, given the CTELS1 construct. The polypeptide chain of this length completely filled the exit tunnel of the ribosome, which was ~100 Å long in eukaryotes and usually accommodated about 30–40 amino acid residues of nascent peptide (for review, see [92]). Thus, the extension enabled the full-length luciferase molecule to be completely folded outside the peptide tunnel, while still being attached to the ribosome. As luciferase folding occurred co-translationally [86,90,91,93], this made the enzyme active right upon completion of its synthesis, before its release from the ribosome. In other words, in this case the luciferase started emitting light prior to completion of translation termination (Figure 1a, CTELS) and thus its activity could be detected even if termination was blocked. In contrast, the original luciferase became active only upon its release (Figure 1a, FLUC) and could not emit light if termination was inhibited. Thus, the continuous measurement of luciferase activity in two parallel translation mixtures, programmed by these constructs, enabled analysis of translation termination rate under variable conditions.

Importantly, the 3′ proximal parts of FLUC and CTELS1 coding region, as well as their 3′ UTRs, are identical, eliminating the possibility that a difference in these regions could produce some effects unrelated to termination. However, such a design complicated the introduction of new sequences into the CTELS1 stop codon vicinity via PCR, restricting its usage for investigation of the effects brought by 3′ UTRs. Thus, we also prepared another variant of the CTELS construct (CTELS2), where the C-terminal extension was represented by a distinct sequence, the last 50 codons of the human β-globin gene (*HBB*) coding region. This variant allowed the production of mRNA derivatives with an altered stop codon vicinity, simply by changing the reverse primer during the preparation of transcription template (see Materials and Methods for details).

To test our concept, we programmed two cell-free translation systems by the first CTELS pair of capped and polyadenylated transcripts (FLUC and CTELS1). Both mRNAs had the same 5′ and 3′ UTRs derived from the rabbit *HBB* (β-globin) and viral SV40 mRNAs, respectively [83]. The short (53 nt) *HBB* leader provided very rapid and efficient translation initiation, which took just a few seconds to start elongation [88,89]. First, we used the standard, commercially available nuclease-treated rabbit reticulocyte lysate (RRL). However, as RRL is known to poorly reconstitute conditions in the living mammalian cells and in some cases it might produce certain artifacts [83,94], we then also used another cell-free system, the S30 extract of Krebs-2 mouse ascite carcinoma cells [83]. In both systems, two mRNAs produced similar curves of luciferase accumulation (Figure 1b,c, left panels), with a little bit lower CTELS1 yield in the case of RRL, likely due to a lower activity, stability or folding of the modified protein in this system. In the reaction programmed with FLUC, enzyme activity first appeared after ~8.0-8.5 min of incubation, which corresponded well to the translation elongation speed of ~3 nt/s previously reported for such systems [88]. However, in both lysates, we observed a lag (~1 min) in product appearance in the case of the CTELS1 construct (Figure 1b,c, right panels). This lag was obviously caused by translation of the additional 50 codons (which should have taken about 1 min) and implied that the termination process did not contribute significantly to the transit time and were not rate-limiting under these conditions. Similar results were obtained in both lysates with another CTELS pair, capped and non-polyadenylated FLUC and CTELS2 transcripts, both supplemented with the *HBB* leader and 60-nt long 3′ UTR with the artificial sequence (agcatc)_10_ (Figure S1). Thus, the translational properties of the CTELS transcripts are robust and do not majorly depend on the cell-free system, identity of the C-terminal extension or 3′ UTR, or on mRNA polyadenylation.

To use CTELS further for investigating translation termination, we had to confirm the ability of extended luciferase to be active before its release from the ribosome, reported earlier [91,95], in our hands. For this, we used the CTELS2 system to prepare two transcripts encoding luciferase with and without extension, but lacking a stop codon (i.e., both transcripts ended with the last sense codon of the coding region, Figure 1d). Then, we compared their translation kinetics with the one produced by the corresponding conventional, stop codon containing mRNAs, the pair of FLUC and CTELS2 transcripts that had the 60-nt long 3′ UTRs (agcatc)_10_. In the absence of stop codon, translation termination could not occur normally. Instead, a special rescue surprise pathway operated with peptidyl-tRNA on the ribosome in this case (for review, see [96,97,98]).

As expected, in the case of FLUC, the lack of stop-codon caused a substantial delay (~2.5 min) in the appearance of luciferase activity, as well as lowered its overall yield (Figure 1e, left). In contrast, the activity of the extended luciferases (CTELS2) appeared exactly in the same time, irrespective of the stop codon presence in the encoding transcripts (Figure 1e, right). This corresponded well with the expected ability of the enzyme with the extension to exhibit its activity while being attached to the ribosome, before termination occurred. A similar situation was observed in the Krebs-2 ascite system (Figure 1f), although in this case the rate of luciferase accumulation from the nonstop FLUC transcript was unexpectedly high. We suggest that the “nonstop” rescue pathway was more active in this system than in RRL. However, even here the delay in luciferase appearance was very remarkable (Figure 1f, left panel). Thus, we concluded that the CTELS system worked properly and could be used for the analysis of translation termination in various cell-free systems.

### 3.2. Examination of Putative Translation Termination Inhibitors Using the CTELS System

**Figure 2:**
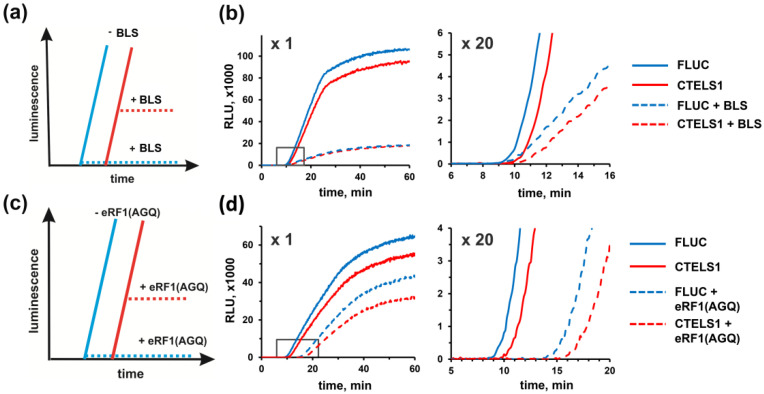
Examination of low- and high molecule weight inhibitors by the CTELS approach. (**a**) Theoretical prediction of blasticidin S (BLS) effects on FLUC and CTELS1 translation, considering the drug specifically inhibits termination. When the compound was added to the cell-free system programmed by FLUC (blue curves), it should suppress its translation due to inhibition of peptide release, while its addition to the mixture with CTELS mRNA (red curves) should not affect the luciferase activity yield at the initial step, until all enzyme molecules synthesized by pioneering ribosomes are completed; only after that CTELS mRNA translation should be also locked, resulting in a plateau in the curve. (**b**) Luciferase activity produced by translation of capped and polyadenylated FLUC and CTELS1 mRNAs in RRL. Representative curves of three independent experiments are shown. (**c**). Theoretical prediction of eRF1(AGQ) effects on FLUC and CTELS1 translation, considering the protein specifically inhibits termination. (**d**) Results of an experiment with the same reporter mRNAs as in (**b**) with addition of 25 ng/μL of the eRF1 mutant (dotted curves) or buffer (solid curves).

We then applied our system to examine the mode of action of some translation termination inhibitors. First, we tested blasticidin S, the widely used small molecule drug targeting both bacterial, archaeal, and eukaryotic ribosomes [44,45,46]. Although this antibiotic was long considered as peptidyl transferase inhibitor, recent structural and biochemical studies provided evidence that in bacteria, blasticidin S largely targets peptidyl-tRNA hydrolysis during termination, rather than peptide bond formation [53,54]. We reasoned that if the drug specifically inhibits termination in the eukaryotic system as well, we should see the differential effects of its addition on the translation of the CTELS mRNA pair (Figure 2a)—luciferase production from the FLUC transcript should be blocked right from the beginning, while CTELS mRNA should initially produce the enzyme at a normal rate, until a pioneer round of its translation is completed. Thus, we added blasticidin S to RRL programmed by the FLUC and CTELS1 transcripts in a concentration, which induced a clear decrease in the luciferase yield (Figure 2b). However, no differential effect on the translation of two mRNAs was detected (Figure 2b, right panel). The result was the same for both lower and higher blasticidin S concentrations (data now shown). We concluded that in eukaryotic systems, at least in those we used here, blasticidin S primarily did not significantly affect termination, but most likely inhibited peptide bond formation during elongation (as was initially proposed for this antibiotic, see [44,46] for review).

We then decided to analyze the effects of the eRF1 derivative with a single amino acid substitution in the GGQ catalytic loop [5,6], the eRF1(G183A) mutant (hereafter called eRF1(AGQ)), in our system. This protein was unable to hydrolyze peptidyl-tRNA, but it could still bind to the ribosome [5]. This turned this protein to a dominant-negative inhibitor of eRF1 and, therefore, it should have affected termination. In this case, we predicted the same effect in the CTELS system as suggested before for blasticidin S (Figure 2c). However, the addition of recombinant eRF1(AGQ) protein to RRL caused unexpected effects, incompatible with the inhibition of termination stage. In particular, it did not strongly inhibit the overall luciferase yield from any reporter, but instead caused a large delay in product appearance (Figure 2d and Figure S2a). Importantly, the delay was detected for both mRNA species and was roughly proportional to the CDS length. Such unusual inhibition mode resembled that of a reduced K^+^ concentration, which is known to slow elongation [88,99]. As the eRF1–eRF3 complex shared its binding site on the ribosome with elongation factors [14,15,16,18], when taken in elevated concentrations, it could compete with eEF1A–tRNA. It should be noted that the concentration of endogenous eRF1 is usually limited in mammalian cells [100], so the addition of recombinant protein to the lysate can induce non-specific eRF1-eRF3 binding to stop-resembling sense codons like UGG [101] or rare codons [102]. In this regard, a non-productive binding of the defective eRF1(AGQ)-eRF3 to elongating ribosomes quite expectedly slows elongation.

In agreement with this possibility, when the same amounts of wt eRF1 were added to the system, we observed not only the delay, but also a severe reduction in the overall luciferase activity (Figure S2b). It should be noted that in our experiments we used concentrations of eRF1 not greatly exceeding those of the endogenous protein. We used ~25–50 ng/μL of recombinant factors, while ~50 ng/μL of the endogenous eRF1 was present in the Krebs-2 system, according to a previous estimation [23], and a similar eRF1 concentration (~85 ng/μL) was reported for HeLa cells [103]. It is likely that in the case of wt eRF1, its non-specific binding to elongating ribosomes caused premature termination, in accordance with the previous findings [102,104].

We concluded that both the wt eRF1 and the eRF1(AGQ) mutant should be used with caution for investigating translation termination, at least in vitro. It should be noted, however, that although the primary and most pronounced affects of eRF1(AGQ) were related to the retarded elongation, we also observed a slight termination-specific effect, which was much more pronounced in the case of the pair of non-polyadenylated FLUC and CTELS2 reporters—in this case, inhibition of FLUC translation was a little bit stronger (Figure S2c).

### 3.3. Short 3′ UTR Inhibits Translation Termination, as Revealed by CTELS

We further applied the new system to investigate to what extent cis-acting factors can affect translation termination rate. It is known that short 3′ UTR can significantly decrease translation efficiency [105,106]. It is logical to assume that this effect is caused by a hindered termination, associated with inappropriate fixation of the short 3′ UTR in the mRNA entry channel. However, this issue has never been examined directly due to the lack of a proper approach, so other explanations are also possible.

**Figure 3:**
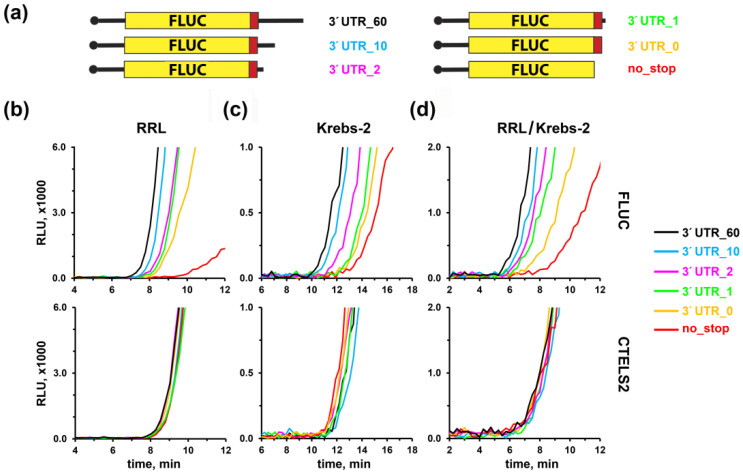
Effects of shortening the 3′ UTR on translation termination, as revealed by the CTELS approach. (**a**) A set of capped non-polyadenylated FLUC mRNA constructs used in the experiment. The same set of CTELS2 transcripts is not shown. (**b**–**d**) Luciferase activity produced by translation of the FLUC and CTELS2 mRNAs in three different cell-free systems: RRL (**b**), Krebs-2 S30 extract (**c**), and the hybrid RRL/Krebs-2 system (**d**). Representative curves of at least three independent experiments are shown.

To systematically investigate the effects of short 3′ UTR on translation termination, we used the CTELS2 system to prepare 5 pairs of stop-codon containing non-polyadenylated transcripts with various 3′ UTR lengths, ranging from 0 to 60 nt (Figure 3a). We also took the mRNA pair with no stop codon, as described above. Translation of these 12 constructs in RRL revealed pronounced delays in the appearance of luciferase activity, depending on the 3′ UTR length, in the case of FLUC transcripts (Figure 3b). However, for all CTELS2 derivatives, the activity arose roughly at the same time (Figure 3b, lower panel). This indicated marked problems occurring specifically at the termination stage. Although the termination failure in cases of the FLUC transcripts with short 3′ UTRs was much less dramatic than with the construct lacking stop codon, the delay was still clear even for the transcript having the 10-nt 3′ UTR. A similar pattern of termination failure was detected for the Krebs-2 S30 extract (Figure 3c), although in this system the nonstop luciferase transcript produced luciferase after a shorter delay, as evident from its comparison with FLUC mRNA having zero-length 3′ UTR. It probably reflected the presence of more efficient “nonstop” rescue pathway in Krebs-2 cells, as suggested above. Finally, we repeated the experiment in the RRL/Krebs-2 hybrid lysate, since such chimeric in vitro systems combine the best features of both their components—high translation efficiency of RRL and relevance to living cells, inherent to the extracts prepared from cultured cells [94]. This hybrid system reproduced the effects observed above (Figure 3d), showing aggravated termination defects with the decreasing 3′ UTR length. Thus, we concluded that reproducible translation termination defects caused by insufficient 3′ UTR length are well documented with the CTELS approach.

### 3.4. Effects of a Read-through Inducing Sequence on Translation Termination Rate

One of the most exciting issues related to translation termination is the phenomenon of stop codon read-through. Manipulating the efficiency of this process could alleviate the symptoms of many hereditary diseases caused by nonsense mutations in important genes. The read-through rate could be easily evaluated by the conventional approach, based on a translation of either a reporter gene with PTC or two in-frame reporter genes with a stop codon inserted between them [23,67,68,107]. However, this method could not reveal the mechanistic basis of the read-through events. It is well-known that stop codon identity, its nucleotide context, and adjacent 3′ UTR sequence strongly affects read-through efficiency [33,34,36], in some cases elevating its rate up to 15% [35]. CTELS offers a unique opportunity to compare translation termination occurring at “normal” and “leaky” (read-through-promoting) stop codon contexts. As an example of the latter, we took a derivative of the 3′-terminal region of Venezuelan equine encephalitis virus (VEEV) mRNA that had the UGA stop codon in a “leaky” context (UGA-C), followed by a special secondary structure. This derivative was shown earlier to induce read-through efficiency of up to 24% in a conventional dual reporter assay [34].

**Figure 4:**
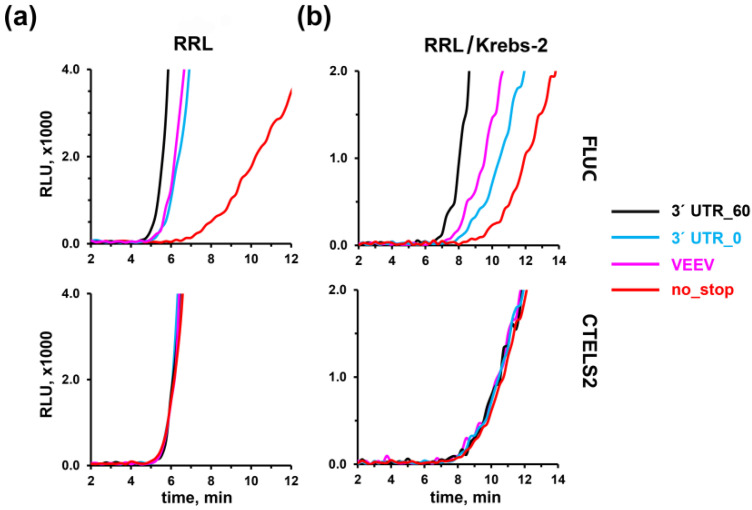
Effects of read-through inducing sequence on translation termination rate. A pair of reporter mRNAs (FLUC and CTELS2) bearing at the 3′ end either a 3′ UTR with the artificial sequence (agcatc)_10_ (3′UTR_60), the structure derived from the VEEV 3′ UTR, or no 3′ UTR at all (3′UTR_0), as well as the nonstop mRNA, were translated in RRL (**a**), or the hybrid RRL/Krebs-2 (**b**) cell-free systems. Representative curves of at least three independent experiments are shown.

We appended FLUC and CTELS2 constructs with 60-nt 3′ UTRs, represented by either the artificial sequence (agcatc)_10_, or the VEEV 3′ UTR derivative, and translated the capped transcripts in RRL or the hybrid RRL/Krebs-2 system (Figure 4). For comparison, the above-described transcripts with zero-length 3′ UTR (with or without stop codon) were translated in parallel.

Our results indicated that the VEEV read-through promoting context caused marked problems at the termination stage, visualized as a delay in FLUC product appearance, similar to that observed in the zero-length 3′ UTR construct (although less severe than in the case of the nonstop mRNA). The effect was well-pronounced in both the RRL and the RRL/Krebs-2 system (Figure 4a,b). It is important to note here again that in the case of FLUC, the luciferase activity could appear not earlier than the first enzyme molecules that were produced [86,91], irrespective of whether these molecules were released as a result of normal or abnormal (delayed) termination. Thus, the observed lag meant that every translation termination event, on 100 percent of mRNA molecules, was hampered (and not only at a portion where read-through event then occurs), as even the fastest terminating ribosomes released the product with the substantial delay. This indicated that “leaky” context caused an intrinsic problem for the terminating ribosome, which entered a long pause, i.e., temporary stalls. We suggest that this stalling could then be resolved by the binding of a non-cognate aminoacyl-tRNA to the A-site, which could lead to incorporation of amino acid instead of peptidyl-tRNA hydrolysis, finally producing the read-through event.

## 4. Discussion

Understanding the molecular basis and mechanisms of translation termination is required for the development of targeted therapy of more than 10% of inherited human disorders caused by nonsense mutations [40]. Despite the availability of biochemical approaches for investigating this process in reconstituted systems [10,82,108], the methods used in vivo or in complete cell extracts are limited to the estimation of read-through efficiency, based on the stop codon containing reporters [67,68,107]. The latter techniques, however, said little about the kinetics and molecular mechanics of the recoding process, as well as posed limitations in the experimental design (e.g., it enabled only limited changes in the sequences, downstream of the stop codon). The recently developed systems-approaches of an in vivo translation analysis, like Ribo-Seq, usually focus on ribosome occupancy of the transcripts and are rarely used to investigate molecular mechanisms and kinetics of protein biosynthesis [109].

Here, we developed a new technique, which utilized a cell-free system in combination with a special mRNA constructs, CTELS. Our approach was based on two luciferase reporters, one of which encoded a C-terminally extended luciferase that is able to emit light before luciferase release from the ribosome (Figure 1a). Comparison of their expression kinetics in cell-free systems allows detecting translation defects specifically attributed to termination. Using CTELS, we unraveled activity of small molecule inhibitors and translation factors, deciphered the effects of 3′ UTR length on termination, and revealed a stalling event at the stop codon in a read-through-provoking sequence context, which probably formed the basis of nonsense suppression.

We offered evidence that blasticidin S, a ribosome-targeting antibiotic that was shown to primarily inhibit termination in bacteria [53,54], mostly affected elongation in eukaryotic systems (Figure 2b). This result was not totally unexpected, as blasticidin S was initially described as a peptidyl transferase inhibitor (for review, see [44]). However, it was nevertheless very important, as blasticidin derivatives were considered to be a promising drug for nonsense-suppression therapy [110].

We further showed that release factor eRF1, as well as its catalytically inactive mutant eRF1(AGQ), when taken in elevated concentration, can perturb and significantly slow elongation, most likely by interfering with the eEF1–tRNA binding to the ribosome (Figure 2d and Figure S2). The ability of release factors to bind non-specifically to near-cognate codons [101] and to induce premature termination at rare codons [102,104] was shown previously by other methods, although this fact is not widely accepted in the field of protein synthesis research. Nevertheless, it is well-known that phenotypes of mutation in eRF1 and eRF3 genes, as well as their overexpression, are pleiotropic and are sometimes unlinked with translation termination defects [111,112].

We then used CTELS for systematically investigating the issue of how 3′ UTR length affects termination. 3′ UTRs are known to substantially affect translation efficiency [113,114]. Although negative effects of short 3′ UTR on the overall translation rate is a widely accepted phenomenon (see, for example, [105,106]), it was not directly linked to a perturbed termination. Indeed, other explanations of this observation are also possible, including different mRNA stability [114], ribosome recycling efficiency [1,13], or closed-loop assisted reinitiation [89]. The new approach allows us to show directly that 0, 1, 2, or 10-nt long 3′ UTRs are insufficient for effective termination (Figure 3). In the first two cases, such inefficiency is most likely explained by an inability to form the proper four-nucleotide U-turn structure in the A-site of the ribosome, which is specific for the pretermination complex [7,8]. Structural studies revealed a base stacking of nucleotides in +4 and +5 positions (relative to the first U of a stop codon), with 18S rRNA bases upon binding eRF1 with the stop codon [7,8,14]. The preference of purines in these two positions is also well-supported by many experimental and bioinformatic approaches (for review, see [13,115]). Moreover, nucleotide identity in some other positions of the mRNA chain 3′ adjacent to a stop codon, including +8, could also affect termination efficiency [33,35,116]. Since up to 10 nt downstream from the A-site codon interact with the ribosome entry channel in bacteria [117] and, most likely, in mammals [118], we reasoned that 10-nt long 3′ UTR could be enough for normal termination. However, our analysis revealed that even in this case a slight, but reproducible delay in FLUC product appearance was observed, as compared to the CTELS2 one (Figure 3). This meant that a protrusion of mRNA portion from the entry channel was likely needed for maximal termination efficiency in a complete translation system, which could be used as a contact with some downstream mRNA-binding factors.

**Figure 5:**
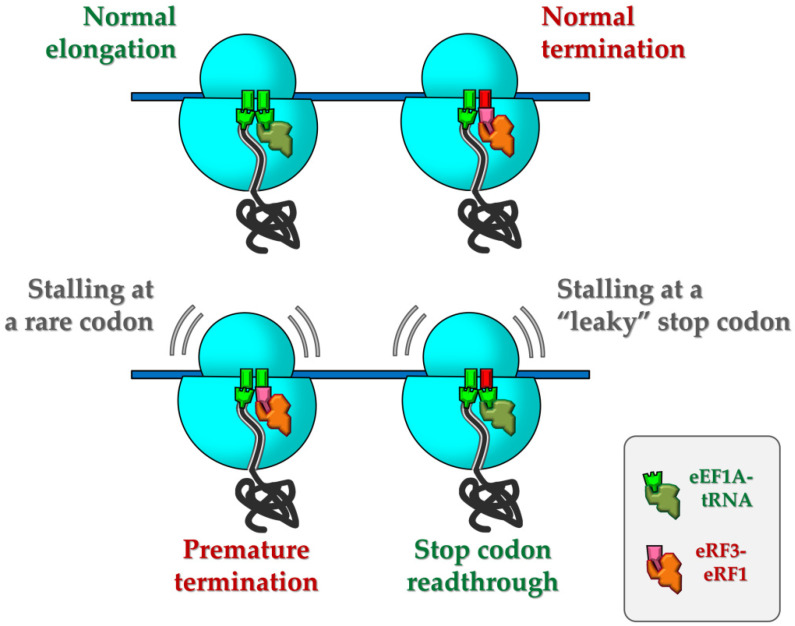
Model of stalling-induced nonsense suppression and premature termination. (Top) Regular elongation and termination that normally occurs at sense (green rectangles) and stop codons (in red), respectively. (Bottom) a rare codon (left) or a read-through-promoting context of stop codon (right) cause temporary stalling, which might be resolved in inappropriate ways due to ribosome binding of release factors to sense codon or a suppressor tRNA to the stop codon, leading to premature termination or read-through, respectively. eEF1A is shown by the dark-green figure, the eRF1–eRF3 complex by the rose and orange ones.

Finally, the new approach allowed us to detect for the first time a temporary stalling during termination at a stop codon in the read-through-promoting sequence context (Figure 4). CTELS showed that in the case of UGA-C followed by the VEEV 3′ UTR, the delay in FLUC product appearance was almost as long as when a transcript with the zero-length 3′ UTR was used. This delay indicated that all 100% of translating mRNA molecules have this pause at the stage of termination, as no luciferase activity appeared earlier. In other words, although only a small portion of ribosomes would finally produce a read-through event on this stop codon, every one of them (even those which would successfully terminate translation) had difficulties during this process. We suggest that this transient stalling, providing a time frame for suppressor tRNA binding, is the intrinsic cause of the following read-through event (Figure 5). Previously, a decoding of the read-through-prone UGA-C stop codon was also shown to occur very slowly in *E. coli* [119], which corresponds well to our finding and suggests the universality of the stalling-induced model of nonsense suppression. Thus, the mechanism of stop codon recoding could be the reversed side of the situation when rare sense codons are recoded by eRF1 (shown in [102,104] and also observed here, see Figure 2d and Figure S2), both are based on transient ribosome stalling followed by its inappropriate resolution.

## 5. Conclusions

Here, we have developed a new in vitro approach, CTELS, which is suitable for detection of translation termination defects and investigation of the kinetics of nascent peptide release. We have shown its applicability by evaluating the effects of small molecule drugs, translation factors, 3′ UTR length and read-through-promoting sequences on the translation termination rate. The results obtained by this technique let us to propose a stalling-induced model of nonsense suppression. We suggest that a modification of the approach could also be applied to study peptide-mediated ribosome stalling, selenocysteine insertion, and other non-canonical events leading to a temporary block of ribosome movement along the mRNA. It might also be used for medium-throughput screenings of read-through drugs for nonsense-suppression therapy.

## Supplementary Materials

The following are available online at https://www.mdpi.com/2218-273X/10/6/911/s1. Figure S1: Time-course of luciferase activity accumulation in a cell-free translation reaction programmed by capped FLUC and CTELS2 transcripts. Figure S2: Effects of recombinant eRF1 (wt and AGQ mutant) on accumulation of luciferase activity in in vitro translation reactions programmed by different reporter mRNAs. Table S1: Plasmids used in this study [83]. Table S2: Primers used in this study.
