# Supplementary material for: CTELS: A Cell-Free System for the Analysis of Translation Termination Rate
Source: Biomolecules. 2020 Jun 16;10(6):911. doi: 10.3390/biom10060911 (PMC7356799; doi:10.3390/biom10060911)
Supplement: Supplementary file 1 [file biomolecules-10-00911-s001.zip › biomolecules-786275-supplementary.docx]

CTELS: A Cell-Free System for the Analysis of Translation Termination Rate

Kseniya A. Lashkevich ^1,†^, Valeriya I. Shlyk ^1,2,†^, Artem S. Kushchenko ^1,3^, Vadim N. Gladyshev ^4^, Elena Z. Alkalaeva ^5^ and Sergey E. Dmitriev ^1,3,5,^*

^1^ Belozersky Institute of Physico-Chemical Biology, Lomonosov Moscow State University, 119234 Moscow, Russia; [akulichksenia@gmail.com](mailto:akulichksenia@gmail.com) (K.A.L.); [ymmo@mail.ru](javascript:void(0);) (V.I.S.); [artkushchenko@gmail.com](javascript:void(0);) (A.S.K.)

^2^ Department of Molecular Biology, Biological Faculty, Lomonosov Moscow State University, 119991 Moscow, Russia

^3^ School of Bioengineering and Bioinformatics, Lomonosov Moscow State University, 119234 Moscow, Russia

^4^ Division of Genetics, Department of Medicine, Brigham and Women’s Hospital, Harvard Medical School, Boston, MA 02115, USA; vgladyshev@rics.bwh.harvard.edu

^5^ Engelhardt Institute of Molecular Biology, Russian Academy of Sciences, 119991 Moscow, Russia; alkalaeva@eimb.ru

***** Correspondence: sergey.dmitriev@belozersky.msu.ru; Tel.: +7-903-2220066

**†** The first two authors contribute equally to this study.

Supplementary materials

**Table 1.** Plasmids used in this study.

| **Plasmid** | **Description** |
| --- | --- |
| pGL3R-β-glo | Original plasmid encoding firefly luciferase flanked by the rabbit *HBB* (β-globin) 5` UTR and SV40 3` UTR [1] |
| CTELS1 | A plasmid encoding firefly luciferase with a C-terminal CDS extension, obtained by repeating the last 150 nt of the luciferase coding region; the plasmid is obtained in this study |
| CTELS2 | A plasmid encoding firefly luciferase with a C-terminal CDS extension derived from the last 150 nt of the human *HBB* gene, appended by the human *HBB* 3` UTR; obtained in this study |

**Table 2.** Primers used in this study.

| **Primer** | **Sequence** |  |
| --- | --- | --- |
| **Primers for plasmid construction** | | |
| FlucExt1F | GAGGTTCCATCTGCCAGGTATC |  |
| FlucExt1R | CGTAGTAACCTGTGATGAATTCCCCACGGCGATCTTTCCGCCCT |  |
| FlucExt2F | GGGAATTCATCACAGGTTACTACGGCGAAAAAGTTGCGCGGAG |  |
| FluExt2R | GAAGGGAGAAAGGCGGACAGG |  |
| GloExtF | CGACTCAATTGTCACGTGGATCCTGAGAACTTC |  |
| GloExtR | AACTTGTTTATTGCAGCTTATAATGGCTGCAATGAAAATAAATGTTTTTTATTAG |  |
| **Primers used for preparation of DNA templates for *in vitro* transcription of reporter mRNAs** | | |
| T7Glo_F | CGCCGTAATACGACTCACTATAGGGACACTTGCTTTTGACACAACTGTG | Forward primer for preparing all DNA templates. T7 promoter is underlined |
| Fla50R | TTTTTTTTTTTTTTTTTTTTTTTTTTTTTTTTTTTTTTTTTTTTTTTTTTAACTTGTTTATTGCAGCTTATAATGG | Reverse primer used for preparation of DNA templates for synthesis polyadenylated transcripts with SV40 3’ UTR |
| NoStopFL_r | gtgCACGGCGATCTTTCCGCCCT | Reverse primers used for preparation of DNA templates for synthesis of FLUC- and CTELS1-based mRNAs with different 3’ UTRs. The annealing parts are shown in capital letters, triplets corresponding to stop codons are in red  - « -  - « -  - « -  - « - |
| 3UTR0FL_r | ttagtgCACGGCGATCTTTCCGCCCT |  |
| 3UTR1FL_r | tttagtgCACGGCGATCTTTCCGCCCT |  |
| 3UTR2FL_r | ctttagtgCACGGCGATCTTTCCGCCCT |  |
| 3UTR10_r | tgctgatgctttagtgCACGGCGATCTTTCCGCCCT |  |
| 3UTR60FL_r | gatgctgatgctgatgctgatgctgatgctgatgctgatgctgatgctgatgctgatgctttagtgCACGGCGATCTTTCCGCCCT |  |
| VEEVrtFL_r | ttcttctttttcctggtcgaggcgcggggcatacgaaatgtatgcacccgcgtcaaaccgtcattgttgttgCACGGCGATCTTTCCGCCCT |  |
| NoStopBG_r | GTGATACTTGTGGGCCAGGG | Reverse primers used for preparation of DNA templates for synthesis of CTELS2-based mRNAs with different 3’ UTRs. The annealing parts are shown in capital letters, triplets corresponding to stop codons are in red  - « -  - « -  - « -  - « -  - « - |
| 3UTR0BG_r | ttaGTGATACTTGTGGGCCAGGG |  |
| 3UTR1BG_r | tttaGTGATACTTGTGGGCCAGGG |  |
| 3UTR2BG_r | ctttaGTGATACTTGTGGGCCAGGG |  |
| 3UTR10BG_r | tgctgatgctttaGTGATACTTGTGGGCCAGGG |  |
| 3UTR60BG_r | gatgctgatgctgatgctgatgctgatgctgatgctgatgctgatgctgatgctgatgctttaGTGATACTTGTGGGCCAGGG |  |
| VEEVrtBG_r | ttcttctttttcctggtcgaggcgcggggcatacgaaatgtatgcacccgcgtcaaaccgtcattgttgttgGTGATACTTGTGGGCCAGGG |  |


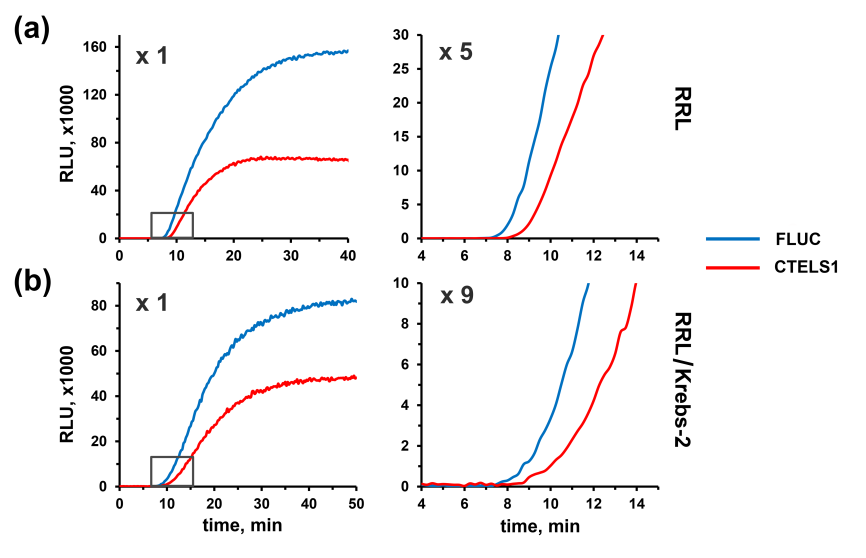


**Figure 1.** Time-course of luciferase activity accumulation in *in vitro* translation reactions programmed by capped FLUC and CTELS2 transcripts in RRL (**a**) and a hybrid RRL/Krebs-2 system (**b**). Right panels represent the boxed areas from the left panels in an increased scale. Representative curves obtained in one out of three independent experiments are shown.


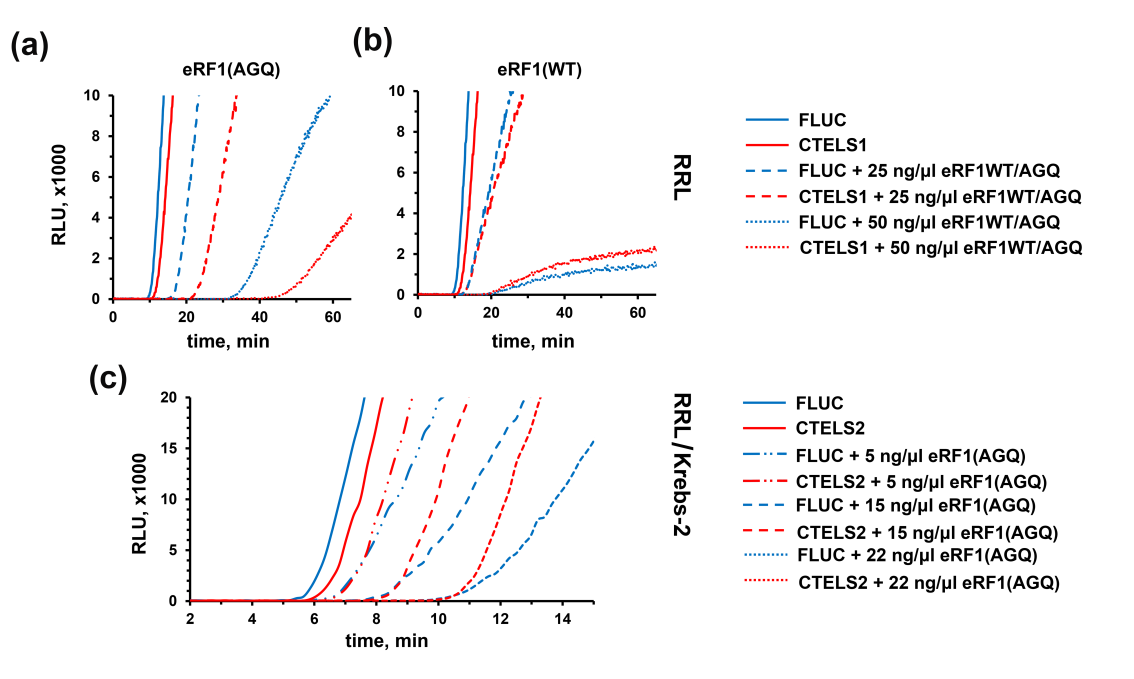


**Figure S2.** Effects of recombinant eRF1 (wt and AGQ mutant) on accumulation of luciferase activity in *in vitro* translation reactions programmed by different reporter mRNAs. (**a**,**b**) Increasing amounts of recombinant eRF1(AGQ) (**a**) or wt eRF1 (**b**) were added to RRL translating capped and polyadenylated FLUC or CTELS1 transcripts. Time-courses of luciferase activity are shown. (**c**) Effects of increasing amounts of eRF1(AGQ) on capped and non-polyadenylated FLUC and CTELS2 transcripts in the hybrid RRL/Krebs-2 system. Representative curves obtained in one out of three independent experiments are shown.
